# Cost-effectiveness of inavolisib plus palbociclib-fulvestrant versus palbociclib plus fulvestrant as first-line treatment in HR^+^/HER2^-^advanced breast cancer

**DOI:** 10.1016/j.breast.2026.104693

**Published:** 2026-01-03

**Authors:** Jiaming Zhu, Ye Ding, Zhengxiong Li, Wen Liu

**Affiliations:** a Fourth Clinical Medical College of Zhejiang Chinese Medical University, Affiliated Hangzhou First People's Hospital, Hangzhou, 310053, PR China; b State Key Laboratory of Natural Medicines, School of Pharmacy, China Pharmaceutical University, Nanjing, 211198, PR China; c NMPA Key Laboratory for Research and Evaluation of Cosmetics, China Pharmaceutical University, Nanjing, 211198, PR China; d School of Medical Informatics and Engineering, Xuzhou Medical University, Xuzhou, 221004, PR China; e School of Humanities and Management, Zhejiang Chinese Medical University, Hangzhou, 310053, PR China

**Keywords:** Cost-effectiveness, PIK3CA mutations, HR^+^/HER2^-^advanced breast cancer, Pharmacoeconomics, Inavolisib

## Abstract

**Purpose:**

This study aims to evaluate the cost-effectiveness of inavolisib plus palbociclib-fulvestrant versus palbociclib-fulvestrant for treating advanced HR^+^/HER2^-^breast cancer patients with PIK3CA mutations from both United States (U.S.) and Chinese healthcare system perspectives.

**Methods:**

A partitioned survival model was used, with survival data from the INAVO120 clinical trials. Primary outcomes assessed were costs, life-years, quality-adjusted life-years (QALYs), and the incremental cost-effectiveness ratio (ICER). Sensitivity analysis was performed to explore the uncertainty of model inputs. Changes in treatment efficacy were modeled to assess their impact on cost-effectiveness. Subgroup analysis was also conducted to refine the findings.

**Results:**

In the base-case analysis, the inavolisib triple regimen showed an ICER of $249,487.22/QALY in the U.S. and $43,812.01/QALY in China, both exceeding their respective willingness-to-pay thresholds. Price simulation indicated that the inavolisib-based combination would be preferred in China if the price were reduced to $133.19/9 mg, while a price of $421.13/9 mg would be required to achieve cost-effectiveness in the U.S. Sensitivity analysis demonstrated robust results from the U.S. perspective, while for China, variations in the price of inavolisib and utility of progression-free-survival influenced the conclusions. Subgroup analyses suggested certain patient subgroups could be cost-effective in the Chinese context.

**Conclusion:**

Compared with palbociclib-fulvestrant, the inavolisib triple regimen is not cost-effective at its current price. A substantial price reduction or careful selection of the target patient may be required for the regimen to be economically viable.

## Highlights

• Inavolisib improved survival outcomes in patients with breast cancer, but was not cost-effective in the U.S. and China.
• Long-term outcomes were projected using partitioned survival models with splines and proportional hazards extrapolations.
• Comprehensive subgroup analyses explored treatment efficacy based on baseline characteristics and prior medication history.
• We employed a dynamic hazard ratio to model the change in drug efficacy over time.

## 1 Introduction

Breast cancer remains the most prevalent malignancy among women. In 2024, the United States (U.S.) and China reported 369,769 and 287,284 newly diagnosed cases, respectively, ranking first and second in terms of incident cancer cases. The mortality rates exceeded 13 % and 20 % in these two countries [1,2], while the 5-year survival rate for patients with distant metastases was only 32 % [3]. Although early screening and prophylactic surgeries have contributed to a declining incidence trend in recent years [3], the persistently high incidence and mortality rates underscore the substantial burden of breast cancer in both nations. Among molecularly stratified subtypes, approximately 70 % of breast cancer patients are HR^+^/HER2^-^ [3], of whom 40 % harbor PIK3CA mutations [4]. Furthermore, breast cancer places a substantial financial strain on healthcare systems, payers, and society, with recurrence-related costs being particularly high [5,6]. Therefore, there is an urgent need for therapies that improve survival outcomes in patients with recurrent breast cancer.

Breast cancer treatment is guided by disease stage and molecular subtype and includes surgery and radiotherapy for local control, as well as systemic therapies such as chemotherapy, endocrine therapy, targeted agents, and immunotherapy. Early-stage patients often receive endocrine therapy; however, one-third develop endocrine resistance and experience disease recurrence, complicating subsequent treatment [7]. For HER2^−^negative patients, the inability to use HER2-targeted antibody-drug conjugates (ADCs) further narrows therapeutic options. Consequently, for endocrine-resistant patients, targeting the PIK3-AKT-mTOR pathway and downstream cell cycle regulators proteins CDK4/6 has become the preferred strategy. Studies demonstrate that PIK3 inhibitors selectively suppress PIK3CA activity, offering clinical benefits for patients with confirmed PIK3CA mutations. In 2019, the first PIK3 inhibitor, alpelisib, was approved in the U.S. While the SOLAR-1 trial reported a clinically meaningful 7.9-month extension in median survival for metastatic breast cancer, this difference did not reach statistical significance. Moreover, 18.1 % of patients discontinued treatment due to severe adverse events (SAEs) [8]. Recently, inavolisib gained approval from both the U.S. FDA and China's NMPA for PIK3CA-mutated, HR^+^/HER2^−^ advanced breast cancer following early-stage endocrine therapy relapse. Notably, while PIK3 inhibitors were previously used primarily in lymphoma, inavolisib is the first PIK3 inhibitor approved in both the U.S. and China for solid tumors. It uniquely inhibits the p110α catalytic subunit of PIK3CA while promoting degradation of mutant p110α. The phase III INAVO120 trial demonstrated that inavolisib in combination with palbociclib and fulvestrant significantly improved survival outcomes compared with palbociclib plus fulvestrant [4] in PIK3CA-mutated, HR^+^/HER2^-^ breast cancer patients with endocrine resistance. The triplet regimen showed significant benefits in survival outcomes, with both overall survival (OS) and progression-free-survival (PFS) reaching statistical significance. After 34 months of follow-up, the inavolisib triplet therapy extended median OS by 7 months (34.0 vs. 27.0 months), prolonged median PFS by 9.9 months (17.2 vs. 7.3 months), delayed the need for chemotherapy by 2 years, significantly improving quality of life. Compared to alpelisib, inavolisib also exhibited a better safety profile, with only 6.8 % of patients discontinuing due to severe AEs. Additionally, oral administration offers advantages over intravenous chemotherapy and ADC, including avoidance of injection-related AEs and improved patient acceptability.

Despite its demonstrated clinical benefits, the high cost of inavolisib poses a barrier to widespread adoption. Policymakers responsible for medical insurance access and pharmaceutical manufacturers must evaluate its real-world value from a cost-effectiveness perspective. Given that the INAVO120 trial enrolled a predominantly U.S. and Chinese patient population, this study will leverage the updated INAVO120 clinical data to conduct the first cost-effectiveness analysis of the inavolisib-triplet regimen versus palbociclib plus fulvestrant for PIK3CA-mutated, HR^+^/HER2^−^ advanced breast cancer in the first-line setting after endocrine resistance. The analysis will adopt U.S. and Chinese healthcare system perspectives, aiming to provide evidence-based insights for pricing negotiations and reimbursement policy decisions.

## 2 Methods

Implementation of the Consolidated Health Economic Evaluation Reporting Standards (CHEERS) 2022 guidelines governed this investigation's reporting quality. Item-by-item validation against the 28-criterion checklist is systematically presented in Supplementary Material Table S1.

### 2.1 Target patient cohort and therapy

Consistent with the phase III INAVO120 clinical study, this research focuses on male and female breast cancer patients with a median age of 54 years who have received endocrine therapy at the early stage and experienced recurrence or disease progression either during treatment or within 12 months after treatment completion. All included patients were confirmed through immunohistochemistry and genetic testing to be HR^+^, HER2^-^, and carrying PIK3CA mutations, with measurable tumor lesions according to Response Evaluation Criteria in Solid Tumors (RECIST) version 1.1. All enrolled patients first underwent genetic testing to confirm PIK3CA mutations, then were randomly assigned in a 1:1 ratio to either the experimental or control group.

In the experimental group, patients received 9 mg of oral inavolisib daily for 28 days, while the control group received a placebo. Both groups took 125 mg of oral palbociclib daily for the first 21 days of each 28-day cycle and received 500 mg of fulvestrant intramuscularly on days 1 and 15 of the first cycle, followed by one injection per subsequent cycle [4]. Treatment continued until radiographic disease progression or unacceptable toxicity. As the clinical trial did not specify follow-up methods, patient monitoring was conducted according to the follow-up recommendations for advanced breast cancer patients in NCCN and CSCO guidelines [9,10]. After disease progression, patients received second-line treatments as listed in the INAVO120 clinical trial, while those who did not undergo subsequent anti-cancer therapy were modeled to receive best supportive care (BSC) by default. The proportions of patients receiving subsequent anti-cancer therapy were listed in Supplementary Material Table S2.

### 2.2 Model construction

**Fig. 1:**
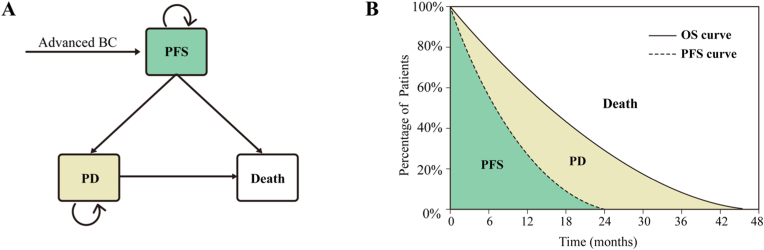
The state transition model (A). and the partitioned survival model (B). Abbreviations: BC, breast cancer; PFS, progression-free survival; PD, progressive disease.

We constructed a partitioned survival model in TreeAge Pro 2022 that stratified all advanced breast cancer patients into three mutually exclusive health states: PFS, progressive disease (PD) and death. PFS probabilities were estimated through integration of the PFS Kaplan-Meier (KM) curve, with PD probabilities determined by subtracting the PFS area from the OS curve area. The number of deceased patients was determined by subtracting those in both PFS and PD states from the total population. The model employed a unidirectional transition structure, allowing patients to move only from PFS to either PD or death states (Fig. 1). The model cycle length was set at 28 days to align precisely with the treatment administration schedule. A 30-year time horizon was implemented to ensure that over 99 % of the cohort would transition to the death state, thereby enhancing the reliability of the cost-effectiveness analysis. Discount rates of 3 % for the U.S. and 5 % for China were applied in accordance with standard health economic evaluation practices [11]. Primary outcomes of the model included total costs, life-years, quality-adjusted life-years (QALYs) and incremental cost-effectiveness ratio (ICER). To account for regional economic variations in drug pricing decisions, willingness-to-pay (WTP) thresholds were established at $150,000/QALY for the U.S [[3], [12]]. times the 2024 per capita GDP as $40,334/QALY for China, in accordance with the methodological standards outlined in the 2020 China Guidelines for Pharmacoeconomic Evaluations [13].

### 2.3 Clinical data

This study obtained OS and PFS KM curves from the INAVO120 phase III clinical trial. Individual patient data (IPD) were extracted from the KM curves using GetData Graph Digitizer software (Version 2.26) and reconstructed IPD using R software (Version 4.3.2). Following recommendations from the UK National Institute for Health and Care Excellence (NICE) and the Canadian Agency for Drugs and Technologies in Health (CADTH), we evaluated seven parametric distributions including exponential, Weibull, Gompertz, gamma, generalized gamma, log-normal, and log-logistic [14] and three flexible models including fractional polynomial (FP), restricted cubic spline (RCS), and Royston-Parmar (RP) spline models for curve fitting and extrapolation. Distributions with higher log-likelihood values and lower AIC were preferred, and final selection was supported by visual inspection of model-predicted curves against KM data. The final survival functions of inavolisib triple regimen and palbociclib- fulvestrant are presented in Table 1, and the goodness-of-fit results are presented in Supplementary Materials Fig. S1 and Table S3.

**Table 1:** Parameters input.

Parameters	Estimate	SA range	Distribution	Reference
**Survival curve parameters**				
OS of inavolisib: RCS2	intercept = −1.55; time 1 = 1.07; time 2 = 0.37			Model fitting
OS of placebo: FP1-2	intercept = −0.94; time = −0.270			Model fitting
PFS of inavolisib: RP-hazard-1 knot = 3	gamma 0 = −3.14; gamma 1 = 0.53; gamma 2 = −1.04; gamma 3 = 1.61; gamma 4 = −0.61			Model fitting
PFS of placebo: RP-hazard-2 knot = 1	gamma 0 = 1.89; gamma 1 = 2.04; gamma 2 = 0.08			Model fitting
**Cost in U.S. ($)**				
*Cost of drug*				
Price of inavolisib per 9 mg	816.68	653.34–980.02	Gamma	RED BOOK [16]
Price of palbociclib per 125 mg	720.09	576.08–864.11	Gamma	RED BOOK [16]
Price of fulvestrant per 500 mg	66.44	53.15–79.73	Gamma	CMS [15]
Capecitabine per 500 mg	0.53	0.42–0.64	Gamma	CMS [15]
Paclitaxel per 100 mg	10.60	8.48–12.72	Gamma	CMS [15]
Alpelisib per 150 mg	425.05	340.04–510.06	Gamma	CMS [15]
Everolimus per 1 mg	7.50	6.00–9.00	Gamma	CMS [15]
Ribociclib per 200 mg	303.18	242.54–363.82	Gamma	CMS [15]
Abemaciclib per150 mg	291.61	233.29–349.93	Gamma	CMS [15]
Drug administration	35.24	28.19–42.29	Gamma	[20]
Cost of BSC per cycle	1,397.29	1,117.83–1,676.75	Gamma	[27]
Cost of terminal care per patient	11,603.15	9,282.52–13,923.78	Gamma	[27]
Genetic alterations screening	3,500.00	2,800.00–4,200.00	Gamma	[28]
Cost of follow up of patients per unit	1,281.72	1,025.38–1,538.06	Gamma	[20]
*Cost of managing AEs (grade>3)* per *event*				
Neutropenia	18,415.85	14,732.68–22,099.02	Gamma	[29]
Thrombocytopenia	24,329.37	19,463.50–29,195.24	Gamma	[29]
Anemia	21,716.14	17,372.91–26,059.37	Gamma	[29]
Hyperglycemia	281.05	224.84–337.26	Gamma	[28]
Stomatitis or mucosal inflammation	10,128	8,102.40–12,153.60	Gamma	[18]
Body surface (m^2^)	1.84	1.47–2.21	Gamma	[17]
Discount rate	0.03	0-0.08	Fixed	[11]
**Cost in China ($)**				
*Price of drug*				
Price of inavolisib per 9 mg	145.43	116.34–174.52	Gamma	yaozh.com
Price of palbociclib per 125 mg	24.18	19.34–29.02	Gamma	yaozh.com
Price of fulvestrant per 500 mg	59.19	47.35–71.03	Gamma	yaozh.com
Capecitabine per 500 mg	0.32	0.26–0.38	Gamma	yaozh.com
Paclitaxel per 100 mg	48.36	38.69–58.03	Gamma	yaozh.com
Alpelisib per 300 mg	285.72	228.58–342.86	Gamma	a
Everolimus per 5 mg	13.41	10.73–16.09	Gamma	yaozh.com
Ribociclib per 200 mg	9.96	7.97–11.95	Gamma	yaozh.com
Abemaciclib per 100 mg	7.19	5.75–8.63	Gamma	yaozh.com
Drug administration	18.08	14.46–21.70	Gamma	[29]
Cost of BSC per cycle	873.95	699.16–1048.74	Gamma	[29]
Cost of terminal care per patient	1,901.59	1,521.27–2,281.91	Gamma	[29]
Genetic alterations screening	266.80	213.44–320.16	Gamma	Local charge
Cost of follow up of patients per unit	166.75	133.40–200.10	Gamma	[29]
*Cost of managing AEs (grade>3)* per *event*				
Neutropenia	413.87	331.10–496.64	Gamma	[29]
Thrombocytopenia	3,410.40	2,728.32–4,092.48	Gamma	[29]
Anemia	510.30	408.24–612.36	Gamma	[29]
Hyperglycemia	3.22	2.58–3.86	Gamma	Local charge
Stomatitis or mucosal inflammation	13.06	10.45–15.67	Gamma	Local charge
Body surface (m^2^)	1.72	1.38–2.06	Gamma	[17]
Discount rate	0.05	0-0.08	Fixed	[11]
*Health-state utilities*				
Progressive disease	0.52	0.42–0.62	Beta	[19]
Progression-free survival	0.85	0.68–1	Beta	[19,21]
Treatment response	0.075	0.06–0.09	Beta	[21]
*Disutility due to AEs (grade>3)*				
Neutropenia	0.13	0.10–0.16	Beta	[30]
Thrombocytopenia	0.11	0.09–0.13	Beta	[31]
Anemia	0.07	0.06–0.08	Beta	[30]
Hyperglycemia	0.01	0.0048–0.0072	Beta	[28]
Stomatitis or mucosal inflammation	0.15	0.12–0.18	Beta	[21]
*Probabilities of AEs in inavolisib group (%)*				
Neutropenia	82.60	66.08–99.12	Beta	[4]
Thrombocytopenia	13.70	10.96–16.44	Beta	[4]
Anemia	6.80	5.44–8.16	Beta	[4]
Hyperglycemia	6.80	5.44–8.16	Beta	[4]
Stomatitis or mucosal inflammation	5.60	4.48–6.72	Beta	[4]
*Probabilities of AEs in placebo group (%)*				
Neutropenia	80.40	64.32–96.48	Beta	[4]
Thrombocytopenia	4.90	3.92–5.88	Beta	[4]
Anemia	1.80	1.44–2.16	Beta	[4]

a Alpelisib is not available in mainland China, its price was substituted with that of Novartis' alpelisib in India.Abbreviations: OS, overall survival; PFS, progression-free-survival; BSC, best supportive care; AE, adverse event; SA, sensitivity analysis.

### 2.4 Cost and utility

This study was conducted from the perspective of healthcare system, focusing solely on direct medical costs, including drug costs, drug management costs, genetic testing costs, BSC, palliative care, and the costs associated with managing SAEs. U.S. drug cost were obtained from the Centers for Medicare & Medicaid Services (CMS) [15], using the manufacturer-reported average sales price (ASP) and Red Book [16]. Drug costs in China were obtained from www.yaozh.com, local fee standards, and published studies. Grade 3–4 AEs were defined as SAEs, and this study only considered the management costs for SAEs occurring in the inavolisib group and the placebo group, with an incidence rate >5 %. The chemotherapy drug dosages used in second-line treatment were computed using body surface area-based measurements, with assumed values of 1.84 m^2^ for the U.S. population and 1.72 m^2^ for the Chinese population [17]. For AEs management costs, due to the lack of data on the cost of oral mucositis treatment in the U.S., the cost for treating infections commonly observed in breast cancer patients was used as a substitute, based on the study by Mistry et al. [18]. The management costs for other AEs were derived from published literature and calculated by multiplying the cost of a single treatment by the reported incidence of the AEs in the INAVO120 clinical trial [4], with costs accounted for only once during the first cycle of the model [19,20]. All costs were adjusted to 2024 U S. dollar (USD) using the Consumer Price Index via https://www.inflationtool.com, and all costs in China were converted to USD using the 2024 average exchange rate of 1$ = 7.1217 RMB.

Utility values are a set of indicators ranging from 0 to 1, used to measure patients' satisfaction with their own health status and their overall sense of well-being. In this study, all utility values were extracted from a study that used the European quality of life five-dimension questionnaire and the standard gamble method to measure health-related quality of life in patients with metastatic breast cancer in the UK [21]. The utility value for PFS was subtracted from the utility decline due to progression to estimate the utility value for PD [19,21,22]. The utility gain was estimated by multiplying the treatment response value reported by Lloyd et al. [21] with the overall response rate of each group in the INAVO120 trial [4]. The utility decline due to AEs was calculated by multiplying the associated disutility by the reported incidence rate of the AEs in the INAVO120 trial. It was assumed that the utility values for the U.S. and Chinese populations were equal (Table 1).

### 2.5 Price simulation

In the U.S., the price of inavolisib was set at $816.68 per 9 mg in the base-case analysis. To account for price fluctuations due to inflation, we varied the unit cost of inavolisib between $0 and $900 and evaluated cost-effectiveness at WTP thresholds of $100,000/QALY and $150,000/QALY. In China, we simulated the impact of varying the unit price of inavolisib within a range of $0 to $300 on the ICER.

### 2.6 Sensitivity analysis

Deterministic sensitivity analysis (DSA) was used to determine the impact of fluctuations in individual input variables on the ICER. The analysis method involved varying each variable by ±20 % from baseline, and discount rates were tested from 0 % to 8 %, relative to the baseline rates of 3 % for the U.S. and 5 % for China [13]. The final results will present the top ten variables with the greatest impact on ICER in the form of a tornado diagram. To assess the model's stability, we conducted a probabilistic sensitivity analysis (PSA) using 10,000 Monte Carlo simulations, introducing random fluctuations within a specified range for all model input variables. In accordance with ISPOR guidelines, variables related to body surface area, drug costs, and AEs management costs were assigned a Gamma distribution, while utility values, AE incidence rates, and proportions of patients receiving different second-line treatments followed a Beta distribution, with 10 % of the baseline value as the standard error range. PSA were presented using incremental cost-effectiveness scatter plots and cost-effectiveness acceptability curves (CEAC).

### 2.7 Scenario analysis

In the base-case analysis, different parametric survival models were applied to the intervention and control groups. To mitigate bias arising from inconsistencies in parametric survival distributions between the two groups, a proportional hazards model was employed. PFS and OS rates for the inavolisib group were estimated based on hazard ratios (HRs) derived from the INAVO120 trial, which reported HRs of 0.67 for OS and 0.41 for PFS for inavolisib compared to the control. We assumed that the HRs remained constant over the 51-month trial follow-up period. However, visual inspection of the Kaplan-Meier curves indicated convergence beyond the follow-up period, suggesting a gradual attenuation of treatment effect over time. Therefore, we modeled a linear convergence of the HRs to 1.0 between month 51 and 10 years, after which the HR was fixed at 1. Additionally, we considered two extreme scenarios: one in which the treatment effect persists indefinitely (HR remains constant lifelong), and another in which the treatment effect ceases immediately after the trial period (HR becomes 1 at month 51). To evaluate the impact of these extrapolation assumptions on the results, the time horizon for the simulation was set to 10 years.

### 2.8 Subgroup analysis

In subgroup analyses, we evaluated patient subgroups based on the following baseline characteristics: age at enrollment, ECOG performance status, menopausal status, presence of visceral disease, liver metastases status, number of metastatic organs, type of endocrine resistance, hormone receptor status, and history of prior endocrine therapy. The ICER and incremental net health benefit (INHB) = IncrementalQALYs−Incrementalcosts/WTP, were calculated using PFS and OS HRs reported in the INAVO120 trial. The HRs were assumed to remain constant over the 51-month trial period and to converge linearly to 1 between month 51 and year 10. WTP thresholds were set at $150,000 per QALY for the U.S. and $40,334 per QALY for China.

## 3 Result

### 3.1 Base-case results

The results showed that in the U.S. and China, the total cost in the inavolisib group was significantly higher than that in the control group, with incremental costs of $255,317.58 and $39,369.85, respectively. Treatment with inavolisib resulted in a gain of 1.13 life-years and 1.02 QALYs in the U.S., yielding an ICER of $249,487.22/QALY. Similarly, in China, inavolisib provided an additional 0.95 life-years and 0.90 QALYs compared to the control, resulting in an ICER of $43,812.01/QALY. Therefore, under WTP thresholds of $150,000/QALY in the U.S. and $40,334/QALY in China, the inavolisib triple regimen was not economically viable as first-line therapy for advanced HR^+^/HER2^-^ breast cancer patients with PIK3CA mutations in both countries.

### 3.2 Price simulation

**Fig. 2:**
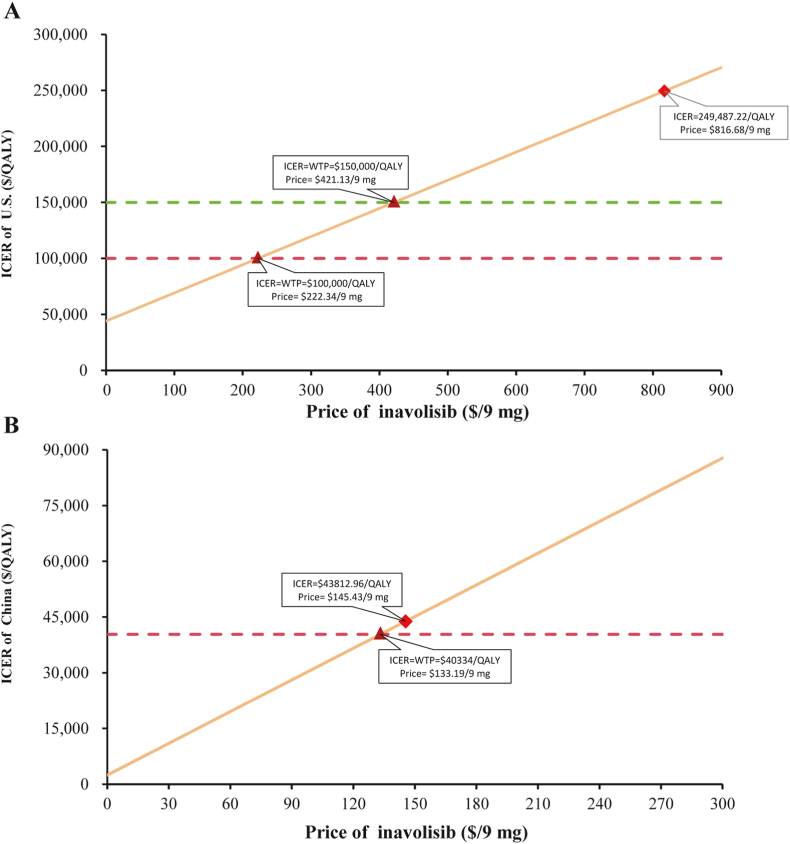
Results of price simulation on ICER in the U.S. (A) and China (B). Abbreviations: WTP, willingness-to-pay; QALY, quality-adjusted life-year; ICER, incremental cost-effectiveness ratios.

The results of the price simulation are shown in Fig. 2. In the U.S., the current price of 9 mg inavolisib is $816.68. When the unit price is reduced to $421.13/9 mg and $222.34/9 mg, inavolisib becomes more economically beneficial at WTP thresholds of $150,000/QALY and $100,000/QALY, with price reduction ratios of 48 % and 72 %, respectively. In China, when the unit price of inavolisib is reduced by 8.4 % ($133.19/9 mg), the inavolisib combination therapy is cost-effective at a WTP threshold of $40,334/QALY.

### 3.3 Sensitivity analysis

**Fig. 3:**
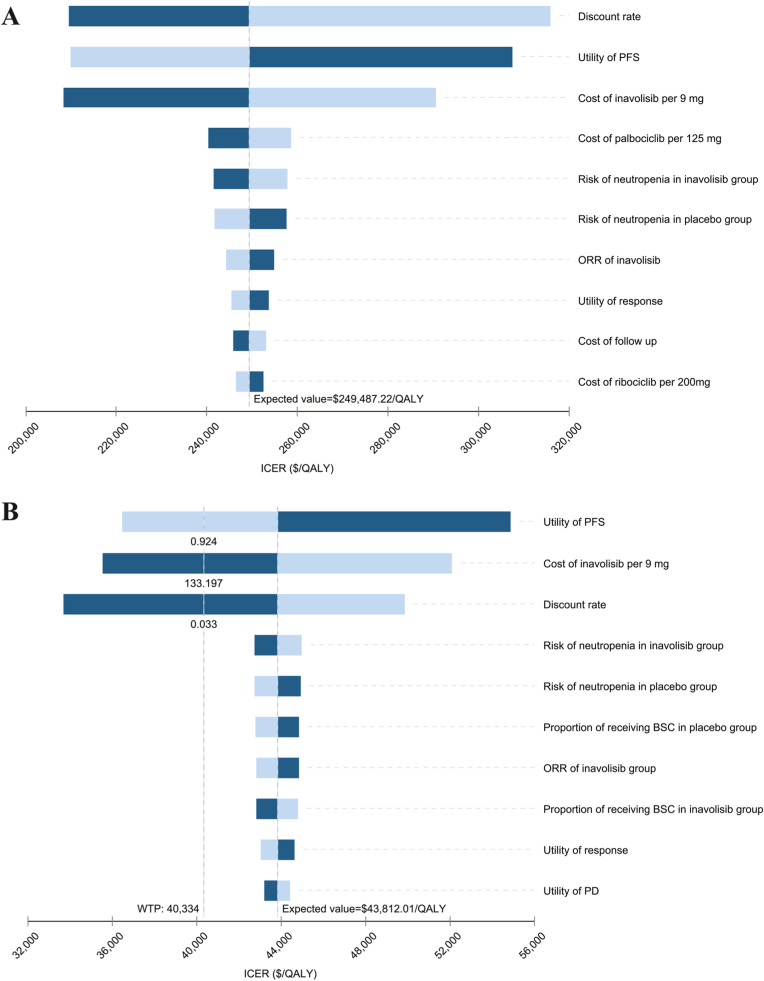
Tornado diagram of deterministic sensitivity analysis of the inavolisib triplet therapy compared to placebo plus palbociclib and fulvestrant in U.S. (A) and China (B). Abbreviations: PFS, progression-free survival; PD, progressive disease; ICER, incremental cost-effectiveness ratio; QALY, quality-adjusted life-year; BSC, best supportive care.

DSA revealed that ICER was most sensitive to the utility value for PFS, the discount rate, and the price of inavolisib in both the U.S. and Chinese settings, with all remaining parameters exerting substantially smaller effects. In the U.S., changes within a ±20 % range for all variables did not reverse the conclusions, the discount rate producing the largest fluctuation in the ICER. In China, the ICER would fall below the WTP threshold if the utility value for PFS exceeded 0.924, the price of 9 mg inavolisib was below $133.19, or the discount rate was lower than 3.3 % (Fig. 3).

**Fig. 4:**
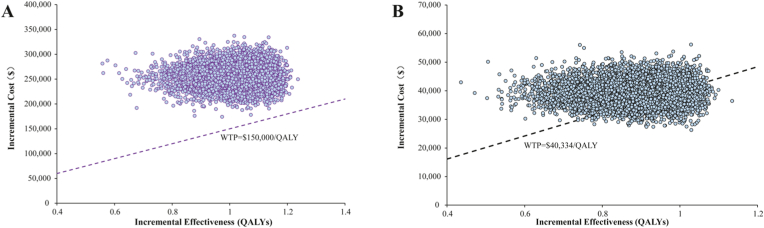
Result of incremental cost-effectiveness scatter plot in U.S. (A) and China (B). Abbreviations: WTP, willingness-to-pay; QALY, quality-adjusted life-year.

**Fig. 5:**
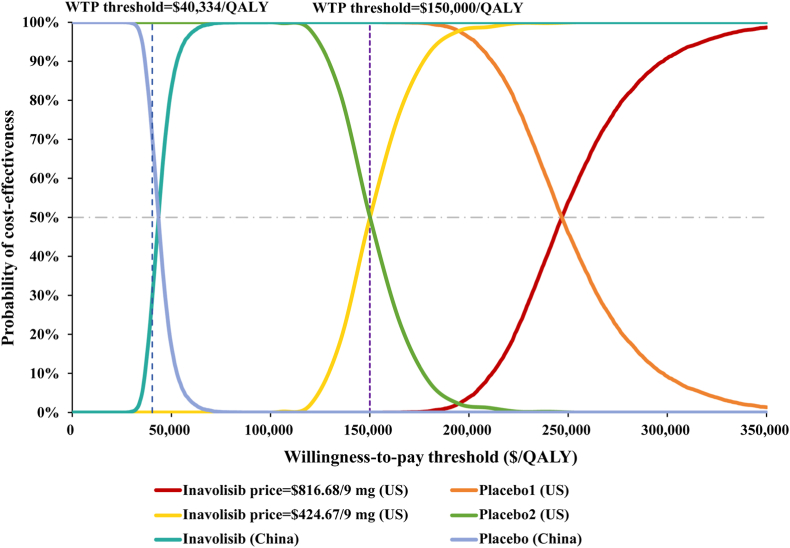
Cost-effectiveness acceptability curve of the inavolisib triplet therapy compared to placebo plus palbociclib and fulvestrant. Abbreviations: WTP, willingness-to-pay; QALY, quality-adjusted life-year.

In U.S., the scatter plot results from the PSA showed that after 10,000 Monte Carlo simulations, all points were above the respective country's WTP threshold, reflecting the model's stability and the robustness of the conclusion that the inavolisib triple regimen is not cost-effective. In China, approximately 29 % of the iterations fell below the WTP threshold, indicating a 29 % probability that inavolisib would be cost-effective at a threshold of $40,334 per QALY under parameter uncertainty (Fig. 4). Fig. 5 presents the CEACs for the U.S. at the current price ($816.68 per 9 mg), a reduced price of $424.67 per 9 mg, and for China at its current price ($145.43 per 9 mg). The results show that at a price of $424.67 per 9 mg in the U.S., the probability of cost-effectiveness reaches 50 % precisely at a WTP threshold of $150,000 per QALY. In China, for the probability of cost-effectiveness to exceed 50 %, a WTP threshold of at least $43,500 per QALY would be required.

### 3.4 Scenario analysis

The results of the scenario analyses are summarized in Table 2. After replacing the survival rate of the control group as the baseline and using the HR to extrapolate the survival of the treatment group, both cost and survival benefits decreased under the assumption that the treatment effect remained constant during the follow-up period but diminished within 10 years. In this scenario, costs decreased by $42,431.68 in the U.S. and by $5,454.94 in China, while QALYs were reduced by 0.12 and 0.06, respectively. Although the ICER in the U.S. remained largely unchanged, the ICER in China fell below the WTP threshold under this assumption and PSA showed that the probability of inavolisib being cost-effective compared to the control exceeded 50 % in China. Under a more conservative scenario where the treatment effect ceased immediately after the follow-up period, costs in both countries remained almost unchanged, while QALYs decreased by 0.4 in the U.S. and 0.31 in China. In this case, the ICERs were substantially higher than the corresponding WTP thresholds, indicating that the inavolisib regimen was not cost-effective. Conversely, under the assumption of a persistent treatment effect, QALYs increased by 0.95 in the U.S. and 0.78 in China compared to the base case, resulting in ICERs of $113,601.74/QALY and $20,708.09/QALY, respectively. In this scenario, inavolisib triple therapy demonstrated cost-effectiveness. Moreover, a simulation with a 10-year time horizon was conducted, in which the ICER increased relative to the base case and did not alter the conclusion.

**Table 2:** Results of the scenario analysis.

Strategy	Cost ($)	LYs	QALYs	Incremental cost ($)	Incremental QALYs	ICER ($/QALY)	Cost-effectiveness probability
**U.S. perspective**							
Base-case: spine model + lifetime horizon							
Placebo	255,539.59	3.00	1.87	–	–	–	–
Inavolisib	510,857.17	4.13	2.89	255,317.58	1.02	249,487.22	0.00 %
Scenario1: adjusted HRs (converge to 1 between 51 and 120 months) + lifetime horizon							
Placebo	255,539.59	3.00	1.87	–	–	–	–
Inavolisib	468,425.48	3.67	2.77	212,885.89	0.90	236,770.82	2.40 %
Scenario 2: adjusted HRs (converge to 1 at 51 months) + lifetime horizon							
Placebo	255,539.59	3.00	1.87	–	–	–	–
Inavolisib	466,512.36	3.40	2.49	210,972.77	0.62	339,884.88	0.00 %
Scenario 3: constant HRs from trial + lifetime horizon							
Placebo	255,539.59	3.00	1.87	–	–	–	–
Inavolisib	479,245.65	4.75	3.84	223,706.06	1.97	113,601.74	95.57 %
Scenario 4: spine model + 10-year time horizon							
Placebo	252,425.73	2.92	1.82	–	–	–	–
Inavolisib	489,304.98	3.61	2.59	236,879.25	0.78	305,399.00	0.00 %
**Chinese perspective**							
Base-case: spine model + lifetime horizon							
Placebo	22,000.38	2.84	1.78	–	–	–	–
Inavolisib	61,370.23	3.79	2.67	39,369.85	0.90	43,812.01	29.22 %
Scenario1: adjusted HRs (converge to 1 between 51 and 120 months) + lifetime horizon							
Placebo	22,000.38	2.84	1.78	–	–	–	–
Inavolisib	55,915.29	3.47	2.62	33,914.91	0.84	40,268.95	51.60 %
Scenario 2: adjusted HRs (converge to 1 at 51 months) + lifetime horizon							
Placebo	22,000.38	2.84	1.78	–	–	–	–
Inavolisib	55,787.88	3.23	2.37	33,787.50	0.59	57,007.03	2.20 %
Scenario 3: constant HRs from trial + lifetime horizon							
Placebo	22,000.38	2.84	1.78	–	–	–	–
Inavolisib	56,700.51	4.31	3.45	34,700.13	1.68	20,708.09	99.7 %
Scenario 4: spine model + 10-year time horizon							
Placebo	21,668.08	2.79	1.74	–	–	–	–
Inavolisib	58,803.33	3.42	2.46	37,135.25	0.72	51,559.52	5.30 %

Abbreviations: HR, hazard ratio; QALY, quality-adjusted life-year; ICER, incremental cost-effectiveness ratio; LYs, life-years.

### 3.5 Subgroup analysis

To evaluate the cost-effectiveness of the inavolisib triple therapy in specific patient populations, we conducted a series of subgroup analyses, with detailed results presented in Table 3. The analyses revealed that patient characteristics significantly influenced the cost-effectiveness of the inavolisib regimen, but conclusions differed between the U.S. and China. In U.S., the INHB was negative across all subgroups evaluated, indicating that the inavolisib combination was not cost-effective in any patient group under current pricing. In contrast, from the China, inavolisib showed potential for cost-effectiveness in certain specific subgroups. Among the subgroups, the INHB was positive for patients who were premenopausal, had one or two metastatic organs, had primary endocrine resistance, and had previously received tamoxifen only. These results suggest that the inavolisib combination represents a cost-effective treatment option in these subpopulations within the Chinese context. In all other subgroups, the conclusion of lack of cost-effectiveness remained unchanged. Among patients aged ≥65 years and those previously treated with aromatase inhibitors (AI) and tamoxifen, the inavolisib regimen was dominated, meaning that it was associated with higher costs and reduced health benefits compared with the control. Furthermore, in the ER+, PR-subgroup, inavolisib may offer limited OS benefit. The ICER in this group was highly sensitive to the HR for OS, suggesting that the economic value of inavolisib decreases rapidly in patients facing a higher risk of mortality. These findings underscore that specific clinical characteristics of patients are critical determinants of the economic viability of inavolisib-based triple therapy in China.

**Table 3:** Results of the subgroup analysis.

Subgroup	Hazard Ratio for OS	Hazard Ratio for PFS	ICER ($/QALY)^a^,^b^	INHB (QALYs)^a^,^b^	Cost-effective probability of inavolisib a,b
Age					
<65 yr	0.65	0.44	242,315.34^a^	−0.54	0.00 %
			41,380.95^b^	−0.02	46.12 %
≥65 yr	1.65	0.96	−268,204.36	−1.16	–
			−55,426.93	−0.92	–
ECOG performance-status at baseline					
0.00	0.69	0.46	259,314.63	−0.59	0.00 %
			44,501.54	−0.07	30.21 %
1.00	0.85	0.58	393,811.12	−0.77	0.00 %
			69,627.70	−0.32	0.20 %
Menopausal status at randomization					
Premenopausal	0.67	0.35	213,532.54	−0.42	1.75 %
			35,956.76	0.11	73.33 %
Postmenopausal	0.81	0.64	413,944.68	−0.80	0.00 %
			74,150.01	−0.36	0.00 %
Visceral disease					
No	1.06	0.43	395,189.75	−0.74	0.00 %
			67,387.06	−0.28	3.22 %
Yes	0.70	0.51	283,392.02	−0.65	0.00 %
			49,556.21	−0.28	11.88 %
Liver metastases at enrollment					
No	0.87	0.56	389,988.54	−0.77	0.00 %
			68,649.81	−0.32	0.10 %
Yes	0.72	0.48	276,034.99	−0.63	0.00 %
			47,602.59	−0.12	20.10 %
No. of organs with metastases at enrollment					
1.00	0.77	0.35	230,278.41	−0.47	1.10 %
			38,505.72	0.04	60.00 %
2.00	0.51	0.47	223,778.75	−0.51	0.30 %
			38,406.62	0.04	66.20 %
≥3	0.86	0.55	375,689.82	−0.76	0.00 %
			65,967.99	−0.30	0.00 %
Resistance to endocrine therapy					
Primary	0.69	0.39	231,588.96	−0.50	0.35 %
			39,103.42	0.03	61.00 %
Secondary	0.77	0.55	329,842.64	−0.71	0.00 %
			57,836.92	−0.24	1.20 %
Hormone receptor status					
ER+, PR–	1.16	0.45	525,871.01	−0.82	0.00 %
			89,924.95	−0.38	0.00 %
ER+, PR+	0.58	0.48	240,731.21	−0.56	0.00 %
			41,441.53	−0.02	43.25 %
Previous endocrine therapy					
AI + tamoxifen	1.15	1.17	−650,894.57	−1.05	–
			−134,803.71	−0.80	–
AI only	0.89	0.62	463,150.53	−0.82	0.00 %
			82,716.31	−0.39	0.00 %
Tamoxifen only	0.68	0.38	215,039.19	−0.43	1.33 %
			38,071.95	0.05	64.98 %

a Upper value: ICER, INHB, and cost-effectiveness probability in the United States.

b Lower value: ICER, INHB, and cost-effectiveness probability in China. Abbreviations: OS, overall survival; PFS, progression-free survival; ICER, incremental cost-effectiveness ratio; INHB, incremental net health benefit; ECOG, Eastern Cooperative Oncology Group; QALY, quality-adjusted life-year; ER, estrogen receptor; PR, progesterone receptor; AI, aromatase inhibitor.

## 4 Discussion

For patients with HR + breast cancer, endocrine therapy, such as AIs or tamoxifen, serves as the cornerstone for controlling estrogen levels. However, a substantial number of patients eventually develop endocrine resistance, wherein tumor cells continue to proliferate despite low estrogen conditions. The aberrant activation of the PIK3/AKT/mTOR signaling pathway plays a critical role in sustaining tumor cell proliferation under these circumstances. Targeting this pathway, a regimen combining inavolisib (a PIK3 inhibitor) with palbociclib and fulvestrant has shown significant clinical benefit. This cost-effectiveness analysis demonstrates that, from both U.S. and Chinese healthcare system perspectives, the inavolisib-based triple regimen yields ICERs of $249,487.22/QALY and $43,812.01/QALY, respectively, compared to palbociclib plus fulvestrant. At the WTP threshold of $150,000/QALY in the U.S., the probability of cost-effectiveness is nearly negligible. In China, at a threshold of $40,334/QALY, the cost-effectiveness probability reaches 29 %. Therefore, although adding inavolisib to palbociclib and fulvestrant provides additional survival benefits for patients with PIK3CA-mutated advanced breast cancer, its high cost raises concerns regarding its economic value and accessibility across different healthcare systems.

Price simulation and DSA indicate that the unit price fluctuation of inavolisib is the primary parameter influencing cost-effectiveness. Enhancing the cost-effectiveness of the inavolisib therapy hinges on the appropriate selection of the treatment population. ER/PR status serves as an important biomarker for predicting the efficacy of this regimen. Specifically, PR status affects treatment outcomes, with the PR-negative subgroup showing no significant OS improvement after inavolisib treatment. This differential efficacy is reflected in the economic evaluation, resulting in a lower ICER in the PR + subgroup compared to the overall population. In the U.S., targeting the ER^+^/PR*^-^*subgroup narrows the gap between the price required for cost-effectiveness and the current list price of inavolisib from $395 to $326. In China, selecting this subgroup increases the probability of the treatment being cost-effective from 29 % to 43 %. A further price reduction of 2.4 % would be required to raise this probability above 50 %. Furthermore, prior treatment with AIs also influences the effectiveness of inavolisib. In clinical practice, AIs are the first-line neoadjuvant treatment for both pre- and postmenopausal HR^+^patients, whereas tamoxifen is often used as an alternative for premenopausal patients. In the U.S., 80 % of breast cancer patients are postmenopausal at diagnosis [3], and most have previously received AIs or a combination of AI and tamoxifen, which is the standard of care recommended by NCCN guidelines [9]. Therefore, stricter patient selection is crucial when considering inavolisib in the U.S. In contrast, standard treatment practices in China differ. According to the CSCO guideline [13], therapeutic strategies strongly depend on menopausal status. Postmenopausal patients are primarily recommended AIs, while the standard for premenopausal patients is tamoxifen with or without ovarian function suppression. Moreover, in China, pre- and postmenopausal patients account for approximately half of breast cancer diagnoses each [23]. Consequently, a larger proportion of patients in China may have received only tamoxifen in earlier lines of therapy than U.S. Thus, inavolisib may be more suitable as a first-line treatment after recurrence in the Chinese context. Additional subgroup analyses suggest that inavolisib provides greater benefit in patients under 65 years of age who were premenopausal at diagnosis, as well as in those with primary endocrine resistance.

In the U.S., alpelisib was the first PIK3 inhibitor approved by the FDA for HR^+^/HER2^-^advanced breast cancer harboring PIK3CA mutations. A recent cost-effectiveness analysis compared alpelisib plus fulvestrant versus fulvestrant monotherapy. The results showed that adding alpelisib to standard care provided an incremental gain of 0.28 QALYs at an additional cost of $94,345.87, yielding an ICER of $340,153.30 per QALY [20]. Consistent with our findings, this study concluded that incorporating a PI3K inhibitor into the standard regimen is not cost-effective within the U.S. healthcare system unless accompanied by appropriate price reductions. This suggests that, regardless of the specific PI3K inhibitor, high drug cost remains a major barrier to widespread adoption in the U.S.

As a novel pharmaceutical agent, inavolisib is under patent protection, which contributes to a pricing structure that is partially decoupled from its actual production costs [24]. An observational study examining the price trends of 32 drugs approved in the U.S. over a 10-year period revealed that drug prices generally decline during the patent exclusivity period, with an average annual net price reduction of 4.7 % after adjusting for inflation [25]. Therefore, it is reasonable to anticipate a natural decrease in the price of inavolisib in the coming years. Furthermore, the UK and Italy have implemented outcomes-based managed entry agreements, which link the reimbursement price of drugs to their real-world clinical performance [26]. Such models help ensure fair returns for high-value therapies while incentivizing manufacturers to focus on long-term treatment outcomes. This approach could serve as a potential reference for the U.S. when considering future pricing strategies for inavolisib, thereby enhancing its cost-effectiveness.

This research has certain constraints. Primarily, the data relied on survival data from the updated INAVO120 Phase III clinical trial. Currently, there is a lack of Phase III study data comparing the triple regimen directly with alternative first-line treatment strategies for HR^+^/HER2^-^ advanced breast cancer patients with PIK3CA mutations and endocrine resistance, precluding direct head-to-head cost-effectiveness comparisons. Second, the utility values applied in this study were derived from the research by Lloyd et al. [21], rather than being extracted directly from the INAVO120 trial. Furthermore, the assumption that utility values for PFS and PD states are identical between the inavolisib treatment group and the standard treatment group may introduce bias. Additionally, rather than adopting utility values specific to the Chinese population, we assumed they were consistent with those from the U.S. However, potential differences in cultural context and health preference valuation between populations may introduce uncertainty into the results. Our study did not account for the disutility of Grade 1–2 AEs. Although this is consistent with previous research and a DSA revealed the ICER was not sensitive to this parameter, this assumption could still potentially lead to an overestimation of the results. Additionally, some second-line treatment and fulvestrant, are administered via intravenous injection, which may lead to drug waste in real-world settings. The impact of this drug waste on cost-effectiveness has not been considered in this analysis. Lastly, the use of inavolisib is dependent on the standardized application of genetic testing technologies and the precise stratification of patients based on their genetic mutations. This highlights the need for a further assessment of the feasibility and affordability of widespread genetic testing in both the U.S. and China.

In the future, efforts should be made to expand genetic testing coverage for advanced breast cancer patients to identify more individuals eligible for inavolisib, thereby making the triple regimen more accessible. Additionally, exploring appropriate adjunctive treatments and reducing the intake of inavolisib per cycle could help control costs and reduce the incidence of AEs.

## 5 Conclusion

From both the U.S. and Chinese perspectives, the ICER for the inavolisib triple regimen is higher than the WTP threshold compared to palbociclib plus fulvestrant, making it not a cost-effective first-line treatment for PIK3CA-mutated HR^+^/HER2^-^ advanced breast cancer. In the U.S., inavolisib would need a price reduction of 48.4 % for the inavolisib combination therapy to become cost-effective. In China, a price reduction of 8.4 % or the selection of an appropriate patient subgroup could make the addition of inavolisib economically viable.

## CRediT authorship contribution statement

**Jiaming Zhu:** Writing – review & editing, Writing – original draft, Project administration, Formal analysis, Conceptualization. **Ye Ding:** Writing – review & editing, Writing – original draft, Formal analysis, Data curation. **Zhengxiong Li:** Writing – review & editing, Project administration, Data curation. **Wen Liu:** Writing – review & editing, Supervision, Conceptualization.

## Ethics approval and consent to participate

Not applicable.

## Consent for publication

Not applicable.

## Funding

This work was supported by the Soft Science Research Program of Zhejiang Province (No. 2024C35060).

## Declaration of competing interest

The authors declare that they have no known competing financial interests or personal relationships that could have appeared to influence the work reported in this paper.

## Data Availability

The data that supports the findings of this study are available in the supplementary material of this article. Further information is available from the corresponding author upon request. (Wen Liu)
