# Supplementary material for: Cost-effectiveness of inavolisib plus palbociclib-fulvestrant versus palbociclib plus fulvestrant as first-line treatment in HR+/HER2-advanced breast cancer
Source: Breast. 2026 Jan 3;85:104693. doi: 10.1016/j.breast.2026.104693 (PMC12809096; doi:10.1016/j.breast.2026.104693)
Supplement: Multimedia component 1 [file mmc1.docx]

Supplementary Materials

**Table S1.** CHEERS Checklist (2022)

| **Topic** | **No.** | **Item** | **Reported?** |
| --- | --- | --- | --- |
| **Title and abstract** |  |  |  |
| Title | 1 | Identify the study as an economic evaluation and specify the interventions being compared. | Yes |
| Abstract | 2 | Provide a structured summary that highlights context, key methods, results, and alternative analyses. | Yes |
| **Introduction** |  |  |  |
| Background and objectives | 3 | Give the context for the study, the study question, and its practical relevance for decision making in policy or practice. | Yes |
| **Methods** |  |  |  |
| Health economic analysis plan | 4 | Indicate whether a health economic analysis plan was developed and where available. | Yes |
| Study population | 5 | Describe characteristics of the study population (such as age range, demographics, socioeconomic, or clinical characteristics). | Yes |
| Setting and location | 6 | Provide relevant contextual information that may influence findings. | Yes |
| Comparators | 7 | Describe the interventions or strategies being compared and why chosen. | Yes |
| Perspective | 8 | State the perspective(s) adopted by the study and why chosen. | Yes |
| Time horizon | 9 | State the time horizon for the study and why appropriate. | Yes |
| Discount rate | 10 | Report the discount rate(s) and reason chosen. | Yes |
| Selection of outcomes | 11 | Describe what outcomes were used as the measure(s) of benefit(s) and harm(s). | Yes |
| Measurement of outcomes | 12 | Describe how outcomes used to capture benefit(s) and harm(s) were measured. | Yes |
| Valuation of outcomes | 13 | Describe the population and methods used to measure and value outcomes. | Yes |
| Measurement and valuation of resources and costs | 14 | Describe how costs were valued. | Yes |
| Currency, price date, and conversion | 15 | Report the dates of the estimated resource quantities and unit costs, plus the currency and year of conversion. | Yes |
| Rationale and description of model | 16 | If modelling is used, describe in detail and why used. Report if the model is publicly available and where it can be accessed. | Yes |
| Analytics and assumptions | 17 | Describe any methods for analysing or statistically transforming data, any extrapolation methods, and approaches for validating any model used. | Yes |
| Characterising heterogeneity | 18 | Describe any methods used for estimating how the results of the study vary for subgroups. | Yes |
| Characterising distributional effects | 19 | Describe how impacts are distributed across different individuals or adjustments made to reflect priority populations. | Yes |
| Characterising uncertainty | 20 | Describe methods to characterise any sources of uncertainty in the analysis. | Yes |
| Approach to engagement with patients and others affected by the study | 21 | Describe any approaches to engage patients or service recipients, the general public, communities, or stakeholders (such as clinicians or payers) in the design of the study. | Not applicable |
| **Results** |  |  |  |
| Study parameters | 22 | Report all analytic inputs (such as values, ranges, references) including uncertainty or distributional assumptions. | Yes |
| Summary of main results | 23 | Report the mean values for the main categories of costs and outcomes of interest and summarise them in the most appropriate overall measure. | Yes |
| Effect of uncertainty | 24 | Describe how uncertainty about analytic judgments, inputs, or projections affect findings. Report the effect of choice of discount rate and time horizon, if applicable. | Yes |
| Effect of engagement with patients and others affected by the study | 25 | Report on any difference patient/service recipient, general public, community, or stakeholder involvement made to the approach or findings of the study | Not applicable |
| **Discussion** |  |  |  |
| Study findings, limitations, generalisability, and current knowledge | 26 | Report key findings, limitations, ethical or equity considerations not captured, and how these could affect patients, policy, or practice. | Yes |
| Other relevant information |  |  |  |
| Source of funding | 27 | Describe how the study was funded and any role of the funder in the identification, design, conduct, and reporting of the analysis | Yes |
| Conflicts of interest | 28 | Report authors conflicts of interest according to journal or International Committee of Medical Journal Editors requirements. | Yes |

From: Husereau, D., Drummond, M., Augustovski, F., de Bekker-Grob, E., Briggs, A. H., Carswell, C., et al. (2022). Consolidated health economic evaluation reporting standards 2022 (CHEERS 2022) statement: Updated reporting guidance for health economic evaluations. *MDM Policy Pract.* 7(1)**,** 23814683211061097. doi:10.1177/2381468321106109

**Table S2** Pre/post-progression therapies: proportion and treatment regimens

| Drugs | Proportion in inavolisib group (%) | Proportion in placebo group (%) | Usage |
| --- | --- | --- | --- |
| Inavolisib | - | - | 9 mg, po, qd |
| Palbociclib | - | - | 125 mg, po, qd, d1-21/28d |
| Fulvestrant | - | - | First week: 500 mg, im, d1 and d15/28d;  then 500 mg, im, d1/28d, Q4W |
| Capecitabine | 31.3 | 33.9 | 1250 mg/m², po, bid, Q3W |
| Paclitaxel | 14.5 | 18.3 | 175 mg/m², iv, 1/21d, Q3W |
| Alpelisib | 6 | 8.3 | 300 mg, po, qd |
| Everolimus | 9.6 | 9.2 | 10 mg, po, qd |
| Ribociclib | 1.2 | 4.6 | 600 mg, po, qd, d1–21/28d, Q4W |
| Abemaciclib | 2.4 | 0 | 150 mg, po, bid |

Abbreviations: po: oral administration; iv: intravenous injection; im: intramuscular injection; sc: subcutaneous injection; qd: once daily; bid: twice daily; Q3W: every 3 weeks; Q4W: every 4 weeks

**Table S3** Goodness-of-fitness results.

|  | OS of inavolisib | | | OS of placebo | | | PFS of inavolisib | | | PFS of placebo | | |
| --- | --- | --- | --- | --- | --- | --- | --- | --- | --- | --- | --- | --- |
| Distribution | LnL | Params | AIC | LnL | Params | AIC | LnL | Params | AIC | LnL | Params | AIC |
| Exponential | -76.52359 | 1 | 155.0472 | -77.95568 | 1 | 157.9114 | -76.22728 | 1 | 154.4546 | -80.87972 | 1 | 163.7594 |
| Weibull | -72.09475 | 2 | 148.1895 | -76.55434 | 2 | 157.1087 | -75.9167 | 2 | 155.8334 | -80.63766 | 2 | 165.2753 |
| Gamma | -72.27382 | 2 | 148.5476 | -76.38647 | 2 | 156.7729 | -75.55148 | 2 | 155.103 | -80.88621 | 2 | 165.7724 |
| Log-Normal | -78.06199 | 2 | 160.124 | -76.96102 | 2 | 157.922 | -73.679 | 2 | 151.358 | -70.60504 | 2 | 145.2101 |
| Gompertz | -73.43459 | 2 | 150.8692 | -77.25828 | 2 | 158.5166 | -76.01431 | 2 | 156.0286 | -77.00367 | 2 | 158.0073 |
| Log-Logistic | -72.05892 | 2 | 148.1178 | -76.47781 | 2 | 156.9556 | -72.52029 | 2 | 149.0406 | -71.55075 | 2 | 147.1015 |
| Generalized Gamma | -72.11005 | 3 | 150.2201 | -76.13717 | 3 | 158.2743 | -73.76608 | 3 | 153.5322 | -69.77313 | 3 | 145.5463 |
| FP1-1 | -74.9923 | 3 | 155.9846 | -77.37764 | 2 | 158.7553 | -75.18999 | 2 | 154.38 | -76.75437 | 2 | 157.5087 |
| FP1-2 | -72.97714 | 3 | 151.9543 | **-75.98662** | **2** | **155.9732** | -75.27226 | 2 | 154.5445 | -81.6331 | 2 | 167.2662 |
| FP2-1 | -69.26428 | 4 | 146.5286 | -77.14043 | 3 | 160.2809 | -74.15805 | 3 | 154.3161 | -76.73936 | 3 | 159.4787 |
| FP2-2 | -68.89391 | 4 | 145.7878 | -77.06589 | 3 | 160.1318 | -73.66038 | 3 | 153.3208 | -75.97246 | 3 | 157.9449 |
| RCS1 | -69.90477 | 3 | 145.8095 | -77.03101 | 3 | 160.062 | -73.63684 | 3 | 153.2737 | -75.64292 | 3 | 157.2858 |
| RCS2 | **-69.08929** | **4** | **146.1786** | -76.69646 | 4 | 161.3929 | -70.94544 | 4 | 149.8909 | -73.63277 | 4 | 155.2655 |
| RP-hazard-1 | -69.11327 | 4 | 146.2265 | -76.55434 | 2 | 157.1087 | **-69.81745** | **5** | **149.6349** | -66.45051 | 5 | 142.901 |
| RP-hazard-2 | -72.09496 | 2 | 148.1899 | -76.07716 | 3 | 158.1543 | -70.03396 | 6 | 152.0679 | **-70.12884** | **3** | **146.2577** |
| RP-odds-1 | -69.00058 | 4 | 146.0012 | -76.47765 | 2 | 156.9553 | -69.4835 | 6 | 150.967 | -66.56849 | 5 | 143.137 |
| RP-odds-2 | -70.25107 | 3 | 146.5021 | -76.48037 | 3 | 158.9607 | -69.90809 | 5 | 149.8162 | -66.71389 | 7 | 147.4278 |
| RP-normal-1 | -70.06227 | 3 | 146.1245 | -76.96102 | 2 | 157.922 | -69.78112 | 6 | 151.5622 | -70.60503 | 2 | 145.2101 |
| RP-normal-2 | -69.48165 | 4 | 146.9633 | -76.28513 | 3 | 158.5703 | -71.33913 | 4 | 150.6783 | -66.67726 | 7 | 147.3545 |

Abbreviations: OS: Overall survival; PFS: Progression-free survival; LnL: Log-likelihood; Params: Parameters; AIC: Akaike information criterion; FP: Fractional polynomial; RCS: Restricted cubic spline models; RP: Royston-Parmar models. Bold data means that this model is the model we finally selected.

(a)


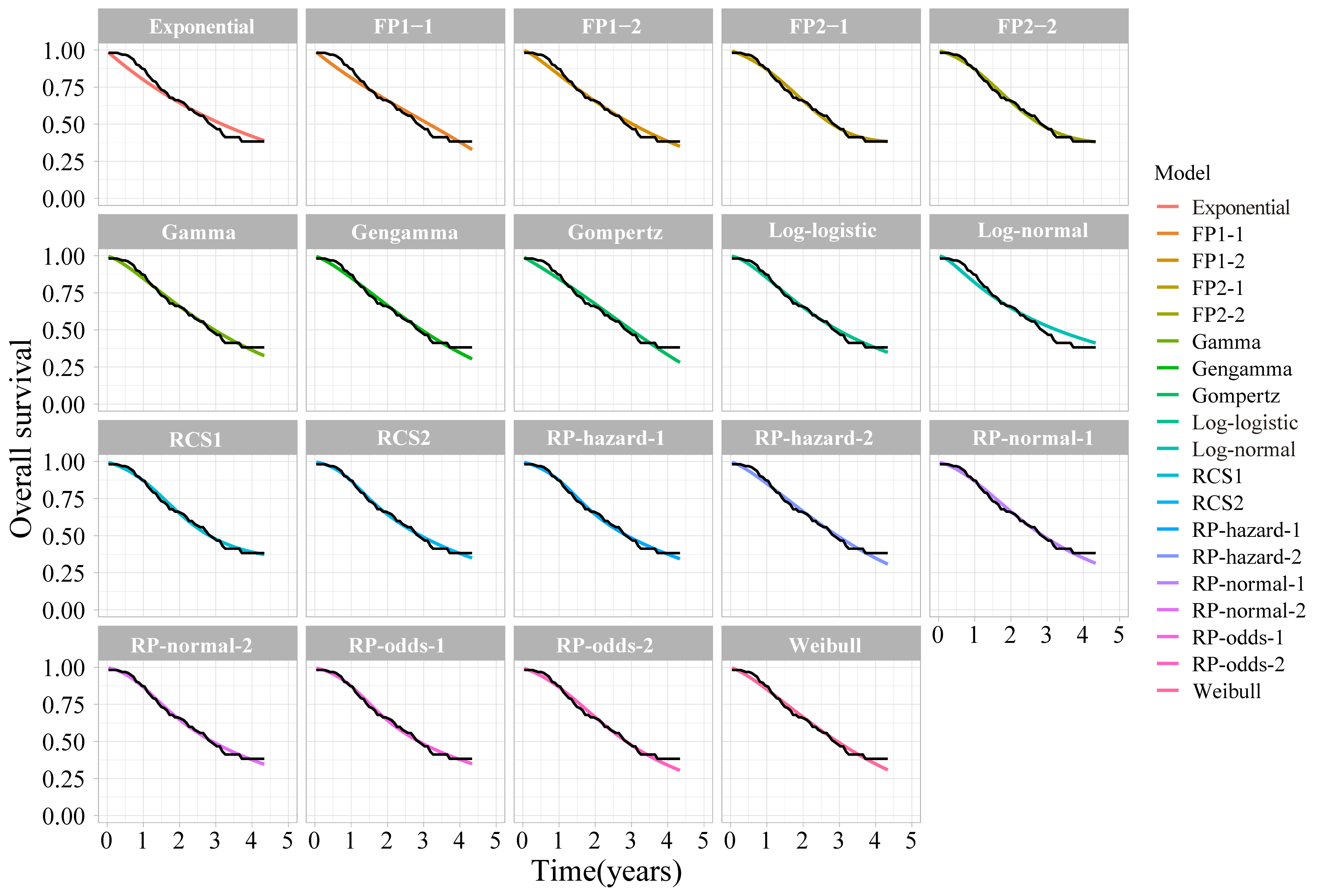


(b)


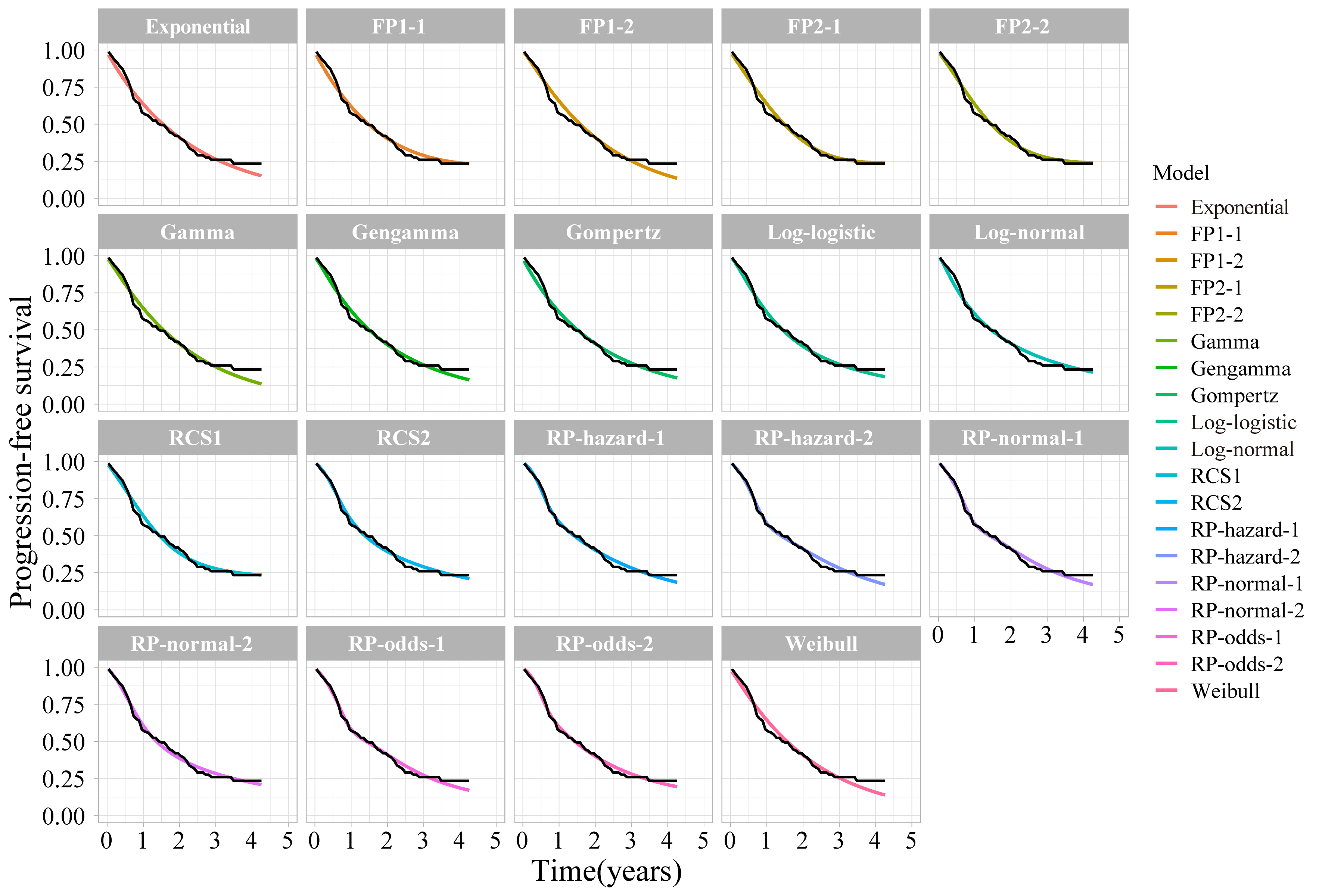


(c)
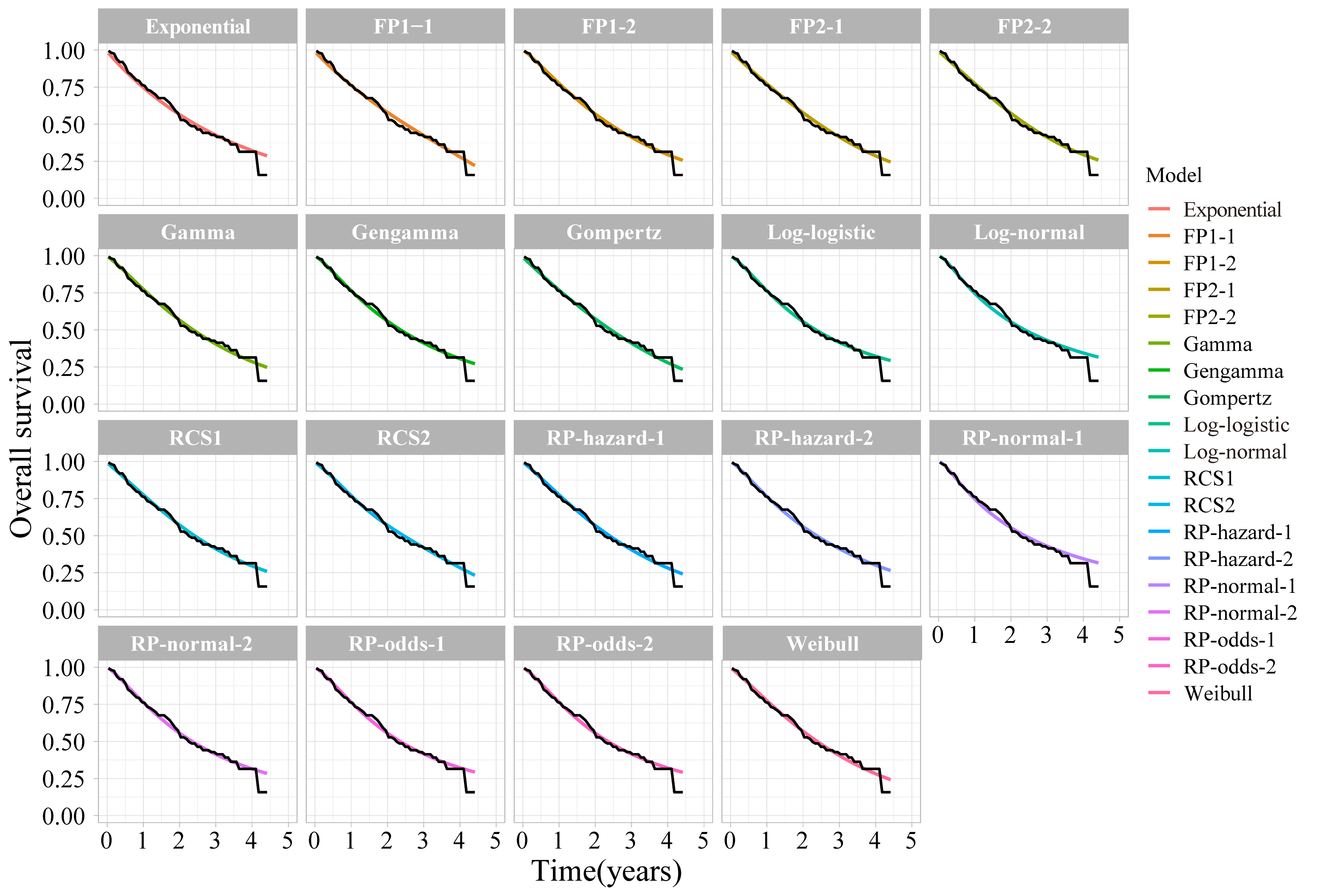


(d)
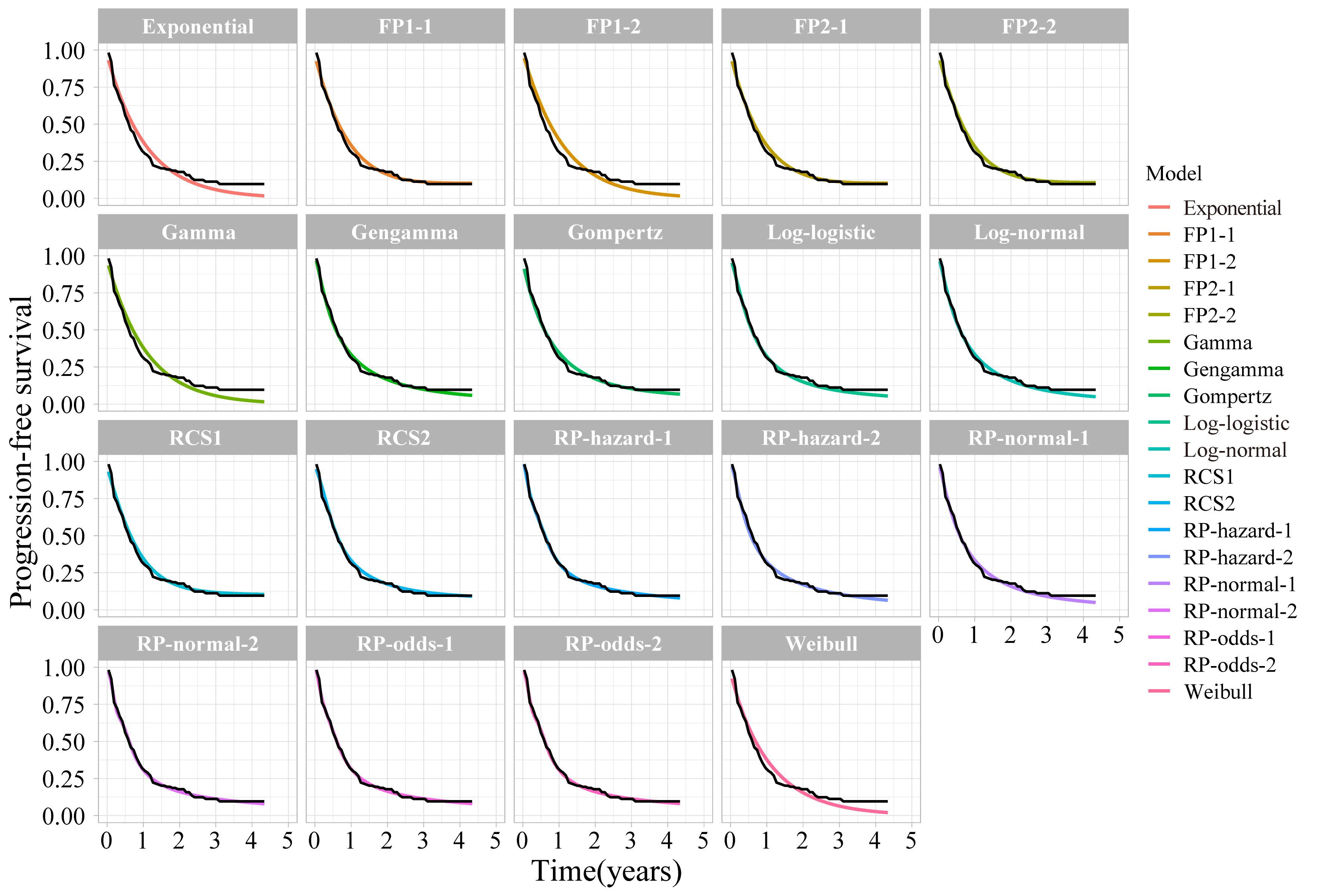


Figure S1 Survival plots showing the goodness-of-fit. OS of inavolisib (a), OS of placebo (b), PFS of inavolisib (c), PFS of placebo (d) Abbreviations: OS: overall survival; PFS: progression-free survival; Black line shows the original KM curves
